# The Role of Plant-Based Diets in Preventing and Mitigating Chronic Kidney Disease: More Light than Shadows

**DOI:** 10.3390/jcm12196137

**Published:** 2023-09-22

**Authors:** Diana Zarantonello, Giuliano Brunori

**Affiliations:** Department of Nephrology, Santa Chiara Hospital, APSS, 38122 Trento, Italy; giuliano.brunori@apss.tn.it

**Keywords:** plant-based diet, chronic kidney disease, nutritional concerns, hyperkalaemia, plant-based meat alternative

## Abstract

Chronic kidney disease (CKD) is a non-communicable disease that affects >10% of the general population worldwide; the number of patients affected by CKD is increasing due in part to the rise in risk factors such as obesity, hypertension, and diabetes mellitus. As many studies show, diet can be an important tool for preventing and mitigating the onset of non-communicable diseases. Plant-based diets (PBDs) are those that emphasize the consumption of plant foods and may or may not include small or moderate amounts of animal foods. Recently, these diets have received increasing interest because they have been associated with favourable effects on health and also appear to protect against the development and progression of CKD. PBDs, which are associated with protein restrictions, seem to offer adjunctive advantages in patients with chronic kidney disease, as compared to conventional low-protein diets that include animal proteins. The principal aims of this review are to provide a comprehensive overview of the existing literature regarding the role of plant-based diets and low-protein, plant-based diets in the context of chronic kidney disease. Moreover, we try to clarify the definition of plant-based diets, and then we analyse possible concerns about the use of PBDs in patients with chronic kidney disease (nutritional deficiency and hyperkalaemia risk). Finally, we offer some strategies to increase the nutritional value of plant-based low-protein diets. In the Materials and Methods section, many studies about plant-based diets and low-protein plant-based diets (e.g., the very-low-protein diet and vegan low-protein diet, LPD) in chronic kidney disease were considered. In the Results and Conclusion section, current data, most from observational studies, agree upon the protective effect of plant-based diets on kidney function. Moreover, in patients with advanced CKD, low-protein plant-based options, especially a very-low-protein diet supplemented with heteroanalogues (VLPDs), compared to a conventional LPD appear to offer adjunctive advances in terms of delaying dialysis and mitigating metabolic disturbances. However, further studies are necessary to better investigate the possible metabolic and cardiovascular advantages of plant-based LPDs versus conventional LPDs.

## 1. Introduction

Chronic kidney disease (CKD) represents a major public health burden that affects >10% of the general population worldwide, amounting to >800 million individuals, and has emerged as one of the leading causes of mortality; predictions suggest that CKD will become the fifth highest cause of years of life lost globally by 2040 [1]. Hypertension, diabetes, metabolic syndrome, and obesity represent the most important risk factors for CKD, and the adoption of a healthy diet may reduce the incidence of these pathologies and prevent damage to the kidneys [2,3,4,5].

It is known that CKD (defined as a reduction in the estimated filtrate rate and/or the presence of albuminuria–proteinuria) increases the risk of death, cardiovascular disease, and hypertension, and predisposes individuals to the progression of kidney disease to end-stage renal disease (ESRD) [5,6].

CKD progression also correlates with the onset of anaemia; metabolic acidosis, which predisposes individuals to hyperkalaemia; the retention of uremic toxins, including phosphorus; and hyperphosphatemia, which contributes to high blood levels of Fibroblast growth factor 23 (FGF23), low levels of 1.25-dihydroxy vitamin D3, and secondary hyperparathyroidism, which leads to mineral bone disease (osteoporosis and a high risk of bone fractures), vascular calcifications, and cardiovascular events that cause a dramatic increase in healthcare costs [6,7,8]. Pharmacological therapies (erythropoietin, phosphate and potassium binders, calcium mimetics, etc.) and renal replacement therapies (haemodialysis, peritoneal dialysis, and renal transplant) that are used for the advanced stages of CKD are expensive [9]. Individuals with advanced CKD are also at high risk of developing a spectrum of nutritional disorders such as undernutrition, protein–energy wasting (PEW), and electrolyte disturbances. Dietary changes represent a low-cost and powerful means of mitigating metabolic abnormalities in CKD and may also help to reduce the pill burden on and healthcare costs incurred by nephropathy patients [7].

Recently, plant-based diets (PBDs) have received increasing interest because they have been associated with favourable effects on health. Moreover, growing evidence suggests that PBDs may have favourable effects on kidney health through primary and secondary prevention.

The principal aims of this review are as follows: (1) to clarify the current definition of plant-based diets; (2) to evaluate if adoption of these diets can be useful in the primary prevention of CKD; (3) to evaluate how plant-based low-protein diets can mitigate the metabolic alterations that occur in non-dialysis-dependent chronic kidney disease (NDD-CKD), and if these diets can postpone dialysis; (4) to analyse concerns about the use of these diets; and (5) to propose strategies to improve the nutritional value of plant-based low-protein diets.

## 2. Definition of Plant-Based Diets

The term plant-based diet is common in literature, but there is no consensus on definition [10]. In fact, PBDs may refer only to vegetarian diets [11,12,13,14,15] or may include diets that are mostly based on plant food but include small amounts of meat and fish [16,17].

The term plant-based diet is common in literature, but there is no consensus on definition.

Vegetarian diets include the strictly vegetarian option, also called a 100% plant-based or vegan diet, which excludes all foods of animal origin, and the lacto-ovo-vegetarian option (which includes small amounts of eggs and/or dairy) [10,18]. Another variant of the vegan diet is the whole food plant-based (WFPB) diet, which derives most of its caloric intake from whole, unprocessed, or only minimally processed carbohydrates, and excludes refined and highly processed foods, such as vegetable oil, sugar, and bleached flours [19].

PBD could also describe diets that are mostly plant-based but include also small amount of animal products. Some examples of these are the flexitarian diet (all kinds of fresh meat are still accepted, but only sporadically) and the semi-vegetarian diet (certain types of meat are excluded, as in the pescatarian diet, which includes only fish, or the pollovegetarian diet, which includes only chicken); on other occasions, flexitarian and semi-vegetarian are used synonymously [10,20,21,22]. In addition, there are other diets that could also be considered PBDs, such as the Mediterranean diet and DASH diet (dietary approaches to stop hypertension), as well as the “Blue Zones” diet (a diet that is followed by the people living in the areas of the world where people live the longest lives, consistently reaching the age of 100) [23,24,25].

An important problem regarding the definitions of PBD is that a clear criterion about the quantity of animal-based products that are allowed is absent; for example, even a lacto-ovo-vegetarian diet, if it is rich in eggs and dairy, should not be defined as plant-based; on the contrary, an omnivorous diet, if it is low in animal products (like the flexitarian diet), may be considered plant-based [10].

Furthermore, the definition of PBD should take into account the intake of healthy versus unhealthy plant-based foods [26]; examples of unhealthy plant food are refined grains, fruit juices, desserts, and potatoes [27]. Moreover, with the growth in the market of unhealthy ultra-processed plant foods, it is possible that the quality of vegetarian dietary patterns will worsen, and that their beneficial effects in terms of health will be reduced [26,28]. This also applies to kidney protection. For example, in a study performed on a sample of about 14,700 middle-aged adults enrolled in the Atherosclerosis Risk in Communities study, the different risks of kidney disease development in relation to different dietary patterns were evaluated; higher adherence to a healthy plant-based diet was associated with a lower risk of CKD, whereas higher adherence to a less healthy plant-based diet was associated with an elevated risk [29]. As we will explain later, these issues (the content of animal foods and the inclusion of processed plant-based foods) are also important aspects to consider in identifying the most appropriate plant-based diet for CKD patients.

## 3. PBDs in Primary Prevention of CKD

According to the Academy of Nutrition and Dietetics’ position, “the appropriately planned vegetarian, including vegan, diets are healthful, nutritionally adequate, and may provide health benefits for the prevention and treatment of certain diseases… These diets are at reduced risk of certain health conditions, including ischemic heart disease, type 2 diabetes, hypertension, certain types of cancer, and obesity… these diets are appropriate for all stages of the life cycle… and are more environmentally sustainable” [30]. The position of the Italian Society of Human Nutrition also confirms that vegetarian diets, including vegan diets, are nutritionally adequate, with a reliable source of vitamin B12 [31].

### 3.1. PBDs for Preventing the Major Risk Factors for CKD

Diabetes, hypertension, and obesity are major contributors to the global burden of CKD. A recent umbrella review confirms that vegetarian diets in the general population correlate with a reduced risk of diabetes, dyslipidaemia, and ischemic cardiac disease [20]. Switching from an omnivorous diet to a plant-based diet has also proven able, as evidenced by a recent meta-analysis, to significantly reduce blood pressure, more so in vegan diets than in lacto-ovo-vegetarian diets [32]. Plant-based diets also have beneficial effects on body weight, as evidenced by observational studies, which show that the body mass index, in a given population, is generally higher among omnivores and gradually decreases in parallel with the reduction in the consumption of animal products and in those who follow a plant-based diet, reaching the lowest values among those who follow a vegan diet [33,34]. Similarly, numerous interventional studies have demonstrated the efficacy of the plant-based diet, which is stronger in vegan diets, in reducing body weight through various possible mechanisms, including a reduced caloric intake (linked to the increased intake of foods with low caloric density and high fibre content), an improvement in the functioning of the intestinal microbiota, and increased insulin sensitivity [33,35]. In another umbrella review, the vegan diet was found to be effective in reducing body weight [36]. The incidence of diabetes mellitus as well as metabolic syndrome is also halved in those who follow a vegetarian diet, compared to those who follow an omnivorous diet [37,38,39]. Furthermore, in interventional studies, for diabetics, switching to a vegan diet compared to a conventional American diet resulted in improved glycaemic control, weight loss, a reduced need for diabetes medications, and an improved lipid profile [38].

### 3.2. PBDs’ Effects on CKD Risk

With regard to kidney disease, since in industrialized countries the main causes of its onset are currently represented by secondary forms of arterial hypertension, type II diabetes mellitus, and metabolic syndrome [40], there is a biological plausibility linking PBDs with a lower risk of kidney damage because they reduce the risk of the onset of the pathologies that cause it. This was confirmed by a recent observational study carried out on more than 55,000 people, which showed that those who followed a vegan diet had a reduced incidence of chronic kidney disease compared to those who ate an omnivorous diet, and also compared to those who followed a lacto-ovo-vegetarian diet [41].

Similarly, another study, involving a population of nearly 15,000 people enrolled in the Atherosclerosis Risk in Community Study (ARIC) group, showed that high adherence to a plant-based diet correlated with a decreased incidence of chronic kidney disease as well as a reduced decline in kidney function over time [29]. In a recent observational study in Taiwan, 3618 patients with hyperuricemia were recruited, and a vegan diet was associated with a 31% lower risk of CKD [42].

Likewise, adherence to the DASH diet shows a protective effect against the incidence of CKD [43,44]; the same is true of the Mediterranean diet [44,45].

Even in diabetic patients, the lacto-ovo-vegetarian diet and the vegan diet are associated with a lower occurrence of CKD [46]. Unfortunately, there is currently a lack of randomized controlled trials (RCTs) to support evidence from observational studies.

In conclusion, we can state that plant-based diets, in particular those that mostly reduce individuals’ intake of animal products (such as the vegan diet), appear advantageous in terms of primary prevention of CKD.

## 4. Dietetic Factors’ and Dietary Patterns’ Effects on Kidney Function and CKD Progression

Epidemiological evidence shows that the risk of CKD in the general population may be associated with different dietetic factors.

### 4.1. Protein Intake (Amount and Source)

It has long been known that the intake of protein-rich food and animal protein leads to elevated intraglomerular pressure and chronic glomerular hypertension, and it may have negative consequences for kidney health, especially if the number of nephrons is already reduced or if there are other risk factors for kidney disease, such as hypertension and diabetes [46,47]. In support of this, a Korean general population study reported an association between higher total protein intake and faster estimated glomerular filtration rate (eGFR) decline [48]. Higher protein intake in patients with diabetes was found to be positively associated with a greater decline in eGFR [49].

The type of protein, not just the quantity, appears to be important for the kidney health. A recent study of the general population confirmed an association between higher intake of total animal proteins and proteins from fish, poultry, red meat, offal, and processed meat and higher creatinine levels (and, consequently, lower estimated kidney function); however, a possible confounder related to muscle mass metabolism cannot be fully excluded [50].

The use of a dietetic pattern with a low carbohydrate and high protein intake could be harmful to kidney health and may, in the long term, be associated with a higher risk of developing CKD [51,52]. The ketogenic diet (at least 70% of calories from fat, and no more than 15% of calories from carbohydrates) may be of interest in the treatment of polycystic kidney disease (PKD) because it seems to be able to reduce the growth of cysts in animal models; however, in the general population this diet may be associated with kidney health risks like increased acid load, dyslipidaemia, and increased risk of nephrolithiasis [53].

Moreover, total and animal proteins are associated with the risk of cardiovascular disease and diabetes, even when adjusting for lifestyle and nutritional factors [54]. One possible explanation for the different impact of plant and animal proteins is that they are consumed with other nutrients and substances that make up the “protein package” (salt, fat, cholesterol, acid load, intestinal toxin precursors, etc.), and this could clarify their relationship with cardiometabolic health effects [54].

Other epidemiological studies clearly show that the intake of proteins and fats of animal origin, with particular emphasis on red and processed meats, represents a risk factor for the development of chronic kidney disease [55,56,57,58]. Moreover, high salt intake is an established risk factor for kidney function decline, as well as high intake of sugar-sweetened beverages (SSB) [57,58,59,60,61].

### 4.2. Dietetic Acid Load

Even a high dietetic acid load (DAL) can increase the risk of developing chronic kidney disease [62]. DAL correlates with intake of sulphur-containing amino acids (methionine and cystine), which can mostly be found in animal protein and lead to the formation of sulfuric acid and hydrogen ions in the body [62].

Diets high in DAL induce a low-grade metabolic acidosis state, which may be associated with the development of metabolic alterations such as insulin resistance, diabetes, hypertension, bone disorders, low muscle mass, kidney stones, hyperuricemia, and non-alcoholic hepatic steatosis [63,64,65]. Plant-based diets and vegetarian diets correlate with a reduction in DAL, and in an observational study vegetarians consumed less protein and phosphorus compared to nonvegetarians, but had a higher intake of magnesium and potassium, leading to more favourable DAL scores [66,67]. In a controlled trial, 45 omnivorous individuals were randomly assigned to a vegan or meat-rich diet; after three weeks, the median potential renal acid load (an indirect index of DAL) was significantly lower in the vegan group than in the meat-rich group. This suggests that a vegan diet is a potential means to reduce DAL [68].

In addition to DAL and glomerular hyperfiltration, other potential mediators of kidney damage from animal protein include phosphate content, gut microbiome dysbiosis, and consequent inflammation [47].

### 4.3. Phosphate

Regarding phosphate, it is known that a chronic excess of dietary phosphate can lead to higher blood levels, and that the homeostatic response (phosphaturia) can increase the risk of kidney function decline, partly due to intraluminal/tubular calcium phosphate particles causing kidney inflammation. Indeed, high dietary phosphate intake and hyperphosphatemia are risk factors in the progression of kidney function decline and are associated with increased cardiovascular disease and mortality risk in the general population [69]. Plant-based diets, which provide phosphate in less bioavailable forms, and avoidance of processed foods containing inorganic phosphate food additives can reduce dietary phosphate absorption [70].

### 4.4. Fibre

With regard to microbiome dysbiosis, a westernized diet that contains high levels of fat and low levels of fibre correlates with worse intestinal health, including impaired intestinal barrier function, which causes leaky gut and low-grade systemic inflammation; this condition can induce or exacerbate various systemic diseases, including kidney disfunction. On the contrary, the dietary fibre contained in plant foods is a nutrient that can protect intestinal barrier function and contributes to the maintenance of a healthy microbiome [71]; plant-based diets help to shift the microbial composition in a favourable manner [72].

In an observational study on a general population, higher fibre intake was correlated with better kidney function, less inflammation, and a reduced risk of mortality [73].

Other epidemiological studies show that consumption of fresh fruit, whole grains, low-fat dairy products, and vegetable proteins such as legumes, soy, and oilseeds appear to be protective factors against the development of kidney disease [5,54,57,58,74,75,76,77].

### 4.5. Dietary Patterns’ Effects on Kidney Disease Progression and Mortality Risk

In patients with chronic kidney disease, several observational studies have shown that high adherence to different healthy dietary patterns rich in plant foods and fibres (Healthy Eating Index-2015; Alternative Healthy Eating Index-2010; alternate Mediterranean diet; DASH diet scores; HeartWise Dietary Habits Questionnaire; plant-based) is associated with a delay in CKD progression and improved survival [5,78,79,80]. Moreover, a diet with a higher proportion of proteins from plant sources is associated with lower mortality and slower decline in kidney function [81,82]. A recent systematic review of four RCT (randomized controlled trials) analysed the effect of a vegetarian diet on kidney function and suggested that a vegetarian diet improves kidney filtration in CKD patients [83] (Table 1).

### 4.6. Amino Acid Intake

Finally, amino acid intake is also correlated with the risk of developing CKD. Higher intakes of L-arginine from animal sources and higher loads of branched-chain, alcoholic, and aromatic AAs (that are predominantly in foods of animal origin) were found to be negative factors for kidney health. Conversely, there was no significant association between total or plant-derived L-arginine intake and the risk of CKD, while higher intakes of acidic AAs, proline, and lower intakes of alkaline AAs and small AAs were related to a decreased risk of CKD [86,87].

### 4.7. Plant-Based Diets’ Effects on Proteinuria

While plant proteins seem less likely to induce glomerular hyperfiltration compared to animal proteins, and this may be beneficial in the management of proteinuria, only a few studies have focused on the effect of the type of protein [88]. Barsotti and colleagues demonstrated that the transition from a mixed normoprotein diet to a vegan LPD (0.7 g/kg/day) was associated with a significant decrease in proteinuria in patients with non-diabetic nephrosis [89]. In the aforementioned study performed in Taipei, which evaluated the association between vegetarian diets and CKD prevalence, a lower prevalence of proteinuria was associated with the vegan group [40]. Regarding the use of soy protein instead of animal protein, there is conflicting evidence on proteinuria [90]. Finally, Piccoli and colleagues found that pregnant CKD patients who followed a plant-based moderately protein-restricted diet had a lower increase in proteinuria from the first to the last check-up before delivery, as well as a lower risk of preterm delivery and small-for-gestational-age babies [91].

## 5. Plant-Based Low-Protein Diets in Medical History

The hypothesis that plant-based diets may be more favourable than animal diets for nephropathy patients seems to emerge from early studies. The oldest contribution is by the Italian doctor Mariano Semmola, who in 1850 studied low-protein nutrition in kidney disease. He compared the effects on the urinary albumin, urea, and albumin excretion of four different diets: Diet A was a mixed diet; Diet B was a meat diet (which contained 600–800 g of boiled meat); Diet C was a vegetarian diet (composed of a soup of greens, tomatoes, bread, and chestnuts); and finally, Diet D was vegetable-based and had a very low urea content and low nitrogen content. Diets C and D, both plant-based, resulted in the most effective reduction in albumin and urea extraction [92]. Likewise, the doctor Franz Volhard in 1918 observed a decrease in body urea nitrogen, amelioration of symptoms, and increased survival following the administration of a vegetarian low-protein diet and normal energy intake [93]. Soon after, in 1934, a doctor named Kempner introduced a vegetable diet containing only rice cooked in water or fruit juice, plus sugar or dextrose for caloric balance; this diet was poor in protein (nearly 20 g/day), low in fat (without milk, dairy products, and oil), and very low in salt content. This diet was shown to reduce blood pressure (even in patients with malignant hypertension) and it was prescribed to patients with CKD [94]. However, this diet was very restrictive and exposed patients to a very high risk of malnutrition. In the following years, diets proposed to treat kidney disease were low in protein, but the use of proteins of animal origin (defined as being of “high biological value”) was preferred, due to the high risk of malnutrition in these patients. These diets included protein-free substitutes (cereals) in order to meet the necessary high caloric intake. The most restrictive diet was the Giordano–Giovannetti diet, which contained 20 g of protein primarily of animal origin (mainly from eggs and dairy) [95,96]. However, after the introduction of essential amino acid and keto analogue (EAA and KA) supplements in the early 1970s, it was possible to propose a very low-protein diet (0.3 g/kg of body weight/day) only of plant origin (the supplemented VLPD); this diet was low in phosphate and low in acid load, and also had a favourable effect on bone osteodystrophy [97]. Afterwards, in 1996, the first application of the vegan (100% plant-based) low-protein diet (0.6 g/kg of body weight/day) was published, wherein the supply of essential amino acids came from a mixture of cereals and legumes (a source of complementary proteins). The positive features of this diet are the high ratio of unsaturated to saturated fatty acids, the absence of cholesterol, and the lower net acid production in comparison with a mixed diet [89]. Moreover, a vegetarian soya-based low-protein diet appears to be correlated with lower protein and phosphate intake and a higher caloric intake than an animal low-protein diet [98]. More recently, a low-protein (0.6–0.8 g/kg of body weight/day), plant-based (almost entirely vegan, only occasional milk and yoghurt are allowed) diet supplemented (with EA and KA) has also been used in pregnant women with kidney disease, with favourable effects on the foetal outcomes [99,100]. Finally, in America, a plant-dominant low-protein diet, more than 50% of which is composed of plant protein (PLADO) [101], has been proposed.

## 6. Possible Advantages of Plant-Based Low-Protein Diets

Dietary–nutritional therapy in the treatment of chronic kidney disease has the important aims of counteracting the accumulation of uremic toxins (urea, phosphorus, and toxins of intestinal origin such as p-cresol, indole sulphate, and Trimethylamine N-oxide- TMAO), metabolic alterations (primary metabolic acidosis and hyperparathyroidism), and maintaining a good nutritional state. It is in fact well known that leaving patients with CKD on a free diet with uncontrolled intake of proteins and other nutrients correlates with the onset of a uremic state, with a consequent reduction in appetite and caloric intake, greater incidence of malnutrition, and increased mortality [102,103]. Uremic toxins may also contribute to the onset of a micro-inflammatory state, more frequent at an advanced CKD5 stage [104].

**Figure 1:**
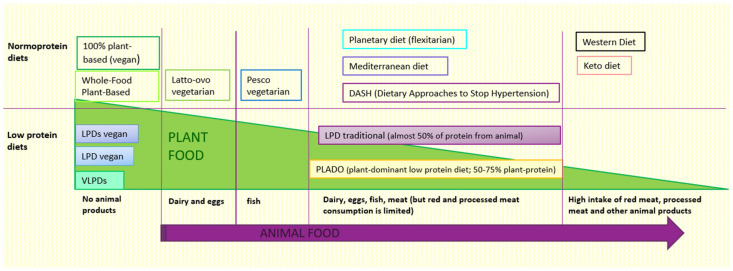
Different types of diet based on the content of animal and plant foods The animal food content in different diets increases in the direction of the purple arrow. In contrast, the plant food content is higher in the diets on the left of the figure and lower in those on the right. LPDs: Low Protein Diet Supplemented. LPD: Low Protein Diet. VLPDs: Very Low Protein Diet Supplemented. Keto: Ketogenic.

In primary prevention of CKD, plant-based diets appear better than omnivorous diets because, as we have seen, they protect against the risk of developing hypertension, diabetes, obesity, and dyslipidaemia [20,21,22,23,24,25,26,27,28,29,30,31,32,33], which are well-known risk factors of kidney disease; some observational studies as well as a recent systematic review have also suggested a protective role against the development and progression of CKD [41,42,43,44,45,83,84,85]. Furthermore, as mentioned before, plant-based diets are more aligned with the nutritional indications of the WHO (Word Health Organization), while the usual western diet is too rich in proteins, sodium, and phosphorus [105]. Therefore, PBDs should certainly be suggested and prescribed in patients at risk of developing CKD or with early kidney disease (stage 1–2 CKD), with an eGFR > 60 mL/min, when guidelines suggest a normal protein intake (0.8 g/kg of body weight/day according to WHO) [106,107]. Generally, PBDs have a normal protein content, but one lower than the protein content of omnivorous diets, which are often characterized by excessive protein intake (Figure 1).

In fact, another important dietary strategy in these patients would be to normalise protein intake, since it is known that in the general population protein intake is normally higher than the suggested amount [108].

### 6.1. Protein Intake (Quantity and Source) in NDD-CKD Stage 3–5

The KDOQI guidelines suggest protein restriction in metabolically stable non-diabetic adults with NDD-CKD stage 3–5, as well as a low-protein diet (0.55–0.6 g/kg of body weight/day) or a very-low-protein diet (0.28–0.43 g/kg of body weight/day) that consists of a vegan diet with EAA and KA supplementation to meet protein requirements (0.55–0.6 g/kg of body weight/day), and protein-free substitutes to reach the required caloric intake. In diabetic adults with CKD 3–5, the KDOQI guidelines suggest a protein intake of 0.6–0.8 g/kg of body weight/day [106]. The KDIGO guidelines recommend a dietary protein intake of 0.8 g/kg/day for diabetic chronic kidney disease patients and a balanced diet high in vegetables, fruits, and whole grains, but low in refined carbohydrates and sugar-sweetened beverages [109]. In contrast, in Italy, a VLPD diet supplemented with EAA and KA (VLPDs) is considered a viable option for selected diabetic CKD patients with CKD stage 5 who are not undergoing dialysis treatments [110]. A recent metanalysis also supported the safety and favourable effect of LPD and VLPD supplemented with KA on diabetic patients with CKD [111].

Experts from the International Society of Renal Nutrition and Metabolism (ISRNM) highlighted that it appears reasonable for clinicians to prescribe the lower end of a streamlined target of 0.6–0.8 g/kg/day protein intake, regardless of the CKD aetiology (diabetic or not). In fact, a lower protein target may be difficult to achieve and dangerous for nutritional status, particularly in those nations where the ketoanalogue supplementation is not available [112].

The KDOQI guidelines do not make recommendations related to the type of protein (animal or vegetable origin) utilized in low-protein diets due to the lack of randomized controlled trials. However, the following ISRNM commentary, in light of the growing number of observational studies, suggests that we should support plant-based protein or eating patterns [112].

### 6.2. RCTs Evaluating the Effects of Vegetable Low-Protein Diets

To reinforce the beneficial effects of vegetable protein, an RCT recently compared 43 patients in LPD with soy protein (60% soy protein + 40% of other vegetable proteins) + KA with 42 patients who received conventional LPD (60% animal protein + 40% of vegetable protein) + KA. It found that LPD + KA with vegetable protein can delay the decrease in eGFR and slow left ventricular hypertrophy. Moreover, the vegetable LPD + KA diet was associated with a slower loss of lean mass and an improvement in phosphorus, urea, and cholesterol levels [113].

There are also RCTs that have compared the traditional low-protein diet or no protein restriction with the supplemented VLPD.

A short observation (4 months) of 24 nephropathy patients that were randomly assigned to a supplemented VLPD or to a conventional LPD showed that the supplemented VLPD maintained nutritional status, improved calcium and phosphorus metabolism, and decreased serum urea nitrogen [114].

In an RCT of older patients (>70 years) with CKD stage 5, a supplemented vegan very-low-protein diet, versus dialysis without protein restriction, delayed dialysis by about eleven months without increased mortality risk and with a reduced risk of hospitalization. The authors also suggest that the supplemented VLPD is a possible strategy to defer dialysis in patients with advanced CKD and good nutritional status who are waiting for angioaccess maturation, or who have a pre-emptive transplantation planned [115].

A prospective, randomized, crossover-controlled trial compared three different nutritional regimes (free diet, Mediterranean diet, and supplemented VLPD) in CKD stage 3b-4 patients. The patients on the MD and supplemented VLPD showed lower diastolic blood pressure and decreased serum levels of urea, sodium, phosphorus, PTH, lysine, and the homocitrulline/lysin ratio (markers of cyanate levels), but showed higher serum levels of bicarbonate and haemoglobin [116].

Garneata and colleagues conducted a randomized controlled trial in 207 patients and showed that the risk of reaching the composite end point (halving the initial eGFR or dialysis initiation) was significantly lower in patients on a supplemented VLPD than in those on a conventional LPD. The authors also observed in vegetarian KA diet patients that the metabolic complications of CKD were clearly improved (serum urea and uric acid were lower, acidosis was corrected, and there was an improvement in mineral metabolism parameters). The authors affirmed that these effects were ascribable not only to KA supplementation but also to the vegetable sources of protein [117].

Finally, a recent systematic review and metanalysis confirmed in a subgroup analysis that a vegetarian very-low-protein diet supplemented with KA (VLPDs) was plausibly more effective than a mixed LPD with KA in slowing the decline in eGFR, and was correlated with improved serum PTH and lowered systolic and diastolic blood pressure [118]. On the contrary, an RCT that compared LPD versus VLPDs found that VLPDs are safe, but do not provide additional advantages to the kidneys or to patient survival; however, a possible bias in this study is that the adherence to dietary prescription was lower in the VLPDs group [119] (Table 2).

### 6.3. Benefits of a Plant-Based Diet for Patients with CKD

We summarize below the documented effects of plant-based diets in mitigating metabolic alteration in CKD:

- First of all, as already mentioned, vegetable proteins have a lower effect on kidney haemodynamics than animal proteins, reducing renal load and hyperfiltration. This effect may act synergistically with the pharmacological effects of RAAS inhibitors and SGLT2 inhibitors [120,121].
- Plant-based low-protein diets have a reduced acid load and counteract the onset of metabolic acidosis; this is because they do not contain animal protein foods (which represent the greatest acid load), and are rich in fruit and vegetables, which have an alkalizing effect [7,105,122]. Therapy with alkali has been shown to slow patients’ progression to end-stage renal disease (ESRD) in randomized controlled trials [123]. An RCT showed that treatment of CKD patients with metabolic acidosis with base-producing fruits and vegetables improves cardiovascular disease risk indicators more effectively than oral sodium bicarbonate [124]. KDOQI guidelines also support prescribing more fruits and vegetables in stage 1–4 CKD-NDD patients in order to decrease body weight, blood pressure, and net acid production [106].
- Plant-based diets have a more favourable lipid composition (low contents of saturated fatty acids and cholesterol), and plant-based low-protein diets can improve the lipid profile in CKD [7,89].
- Plant foods contain phosphorus in the form of phytate, which is poorly bioavailable; consequently, there is less intestinal absorption of phosphate and better control of phosphate levels using fewer drugs [7,125]. An RCT has shown that switching from an omnivorous low-protein diet to a plant-based one reduces blood phosphorus levels after just one week [126]. Moreover, VLPDs, compared to the traditional LPD, can reduce phosphate levels, urinary phosphate, and FGF23 levels [7]. Better correction of hyperparathyroidism with VLPDs also seems to explain the reduced requirement for erythropoietin compared with patients in LPD [127].
- Plant-based low-protein diets (vegan diet 0.7 g/kg/day and VLPDs) provide high amounts of fibres and vitamin K1, which, in association with lower levels of phosphorus and reduced risk of metabolic acidosis, could have a beneficial effect on vascular calcifications and bone health [7]. Moreover, plant-based diets can increase intake of magnesium, which may have additional beneficial effects in counteracting vascular calcifications. In an RCT of patients with CKD stage 3–4, an oral magnesium supplementation significantly retarded the progression of coronary artery calcification [128,129].
- Plant-based diets counteract the state of insulin resistance typical of patients with chronic kidney disease, and improve blood pressure control [130,131].
- Plant-based diets contain lower quantities of carnitine, choline, phosphatidylcholine, tyrosine, and tryptophan, all substances that are metabolized in the gut by the microbial flora, giving rise to uremic toxins (TMAO, p-cresol sulphate, inositol sulphate) with cardio and nephrotoxic properties. People who follow a plant-based diet have lower plasma levels of these substances than omnivores, both in the general population and in nephropathy patients [7,132]. VLPDs can significantly reduce indoxyl-sulphate levels by 72%, and p-cresol sulphate levels by 51%, when compared to a free diet and the Mediterranean diet [133,134]. A prospective randomized controlled crossover study showed that after only 1 week of a VLPD, even preceded by an LPD, CKD patients showed a significant reduction in IS serum levels [133].
- Low-protein plant-based diets are richer in fibres [7,122] with beneficial effects on the intestinal microbiota, counteracting the onset of intestinal dysbiosis and promoting the growth of saccharolytic bacteria and the formation of SCFAs (short-chain fatty acids). SCFAs have a trophic action on the mucosa and strengthen the defence function of the intestinal barrier by counteracting bacterial translocation and the low-grade chronic inflammation typical of nephropathy patients [122,135]. Moreover, fibre intake reduces serum urea levels by promoting a faecal route of excretion for nitrogenous waste, can lower serum levels of AGE (advanced glycation end products), and has a laxative effect (which counteracts the hyperkalaemia risk) [136]. Finally, high fibre intake is known to improve glycaemic control, weight control, and the lipid profile [136].
- Vegetable foods, compared to animal foods, are rich in phytochemicals known to be protective of the cardiovascular system and have a higher capacity to neutralize free radicals (oxygen-neutralized free radicals, ORAC) [40,137]. Patients with CKD in VLPDs show reduced oxidative stress as well as a reduced inflammatory state compared to those in traditional LPD [138]. Moreover, bioactive compounds from plants have been proposed as a nonpharmacological strategy to reduce inflammation and oxidative stress. For example, cruciferous vegetables lead to increased intake of sulforaphane, an agonist of Nrf2. Curcumin reduces Nf-kB, and beetroot, garlic, and berry fruits have antioxidant and anti-inflammatory effects [139].
- Vegetable foods, compared to animal foods, have a lower content of advanced glycation end products (AGEs), even when comparable cooking methods are used [140]. Patients with CKD often have high levels of AGEs because endogenous formation is increased due to oxidative stress, and their renal excretion is reduced [141]. Therefore, a reduced intake of AGEs in the diet may have positive effects, especially in CKD [142].
- Vegetable foods are those with a lower content of “persistent organic pollutants” such as dioxins, furans, polychlorinated biphenyls (PCBs), and organochlorine pesticides, when compared to animal foods. These compounds tend to accumulate in animal fats through so-called ‘bio-accumulation’, and they may be associated with kidney damage [15].
- A vegetarian diet can halve the risk of kidney stones [143].

### 6.4. Quality of Life and Economic Analysis

There are no data about how the different low proteindiets may influence the quality of life (QoL) in nephropathy NDD patients. Two studies have shown that the QoL was not related to the type of diet, but was influenced by age, comorbidity, and setting of care [144,145]. On the contrary, dialysis represents an invasive therapy that reduces QoL and productivity and in the elderly and patients with comorbidities, it may not be tolerated [146]. Therefore, in patients who wish to postpone or avoid dialysis for psychological or clinical reasons (waiting for the maturation of vascular access, a pre-emptive transplant program, or the start of palliative care), VLPDs can counteract uraemic symptoms and delay renal replacement therapy [115]. In some cases, the poor palatability and the high cost of protein-free substitutes can make good compliance difficult, and a vegan LPD may be a better option to improve adherence and satisfaction [89]. Finally, economic analysis also supports the use of a vegetarian VLPD + KA to prevent CKD progression and postpone dialysis as a cost-effective approach, with beneficial effects for patients and healthcare costs [147,148].

## 7. Possible Concerns about Plant-Based Diets in Nephropathy Patients

In the literature, several low-protein diet options made up almost entirely or entirely of vegetables have been described: the low-protein vegan diet, 0.7 g/kg of body weight/day (not supplemented); the low-protein diet, 0.6–0.7 g/kg of body weight/day supplemented with essential amino acids and keto acids (EAA and KA, one tablet per every 10 kg of body weight); the PLADO diet, with 0.6 g/kg of body weight/day per day of protein (at least 70% vegetables); and a very-low-protein diet (0.3 g/kg of body weight/day of protein supplemented with EAA and KA, one tablet for every 5 kg of body weight). Finally, a variant of the PLADO diet for diabetic patients was recently proposed; it is referred to as the PLAFOND diet (plant-focused low-protein for the nutritional management of CKD in diabetes), with 0.6–0.8 g/kg/day of protein with >50% plant-based sources, high dietary fibre, and a low glycaemic index (Table 3) [101,110,149].

Possible concerns have been raised regarding the use of plant-based diets in nephropathy patients.

### 7.1. Amino Acid Deficiency Risk

The first concern is over nutritional adequacy, with particular regard to protein intake and amino acid content.

Plant-based diets often contain less protein than omnivorous diets, but they are not low in protein per se. Analysing the results of the EPIC-Oxford study as well as the French NutriNet-Santé cohort, protein intake is greater in meat-eaters, followed by fish-eaters, followed by lacto-ovo-vegetarians, and finally by vegans, with a percentage of energy intake from protein of about 17.5% for meat-eaters and 13% for vegans [150,151]. These percentages exceed the minimum recommendation of 9% and 10% from the British and American guidelines for energy derived from protein [131]. One exception from this gradient of different protein intake levels in different diet patterns was reported in the Adventist Studies (AHS-2), where the protein intake of lacto-ovo-vegetarians and vegans was strikingly similar to that of fish-eaters, semi-vegetarians, and non-vegetarians. These data are linked to the religious and social characteristics of this population, in which meat-eaters consume little meat and vegans consume lots of protein from legumes and oleaginous seeds; indeed, the median total protein intake in vegans amounted to 14.4% of energy intake, which is quite high when compared to other vegan populations [152]. Moreover, a meta-analysis of nitrogen balance studies found no significant difference in protein needs due to the source of protein being consumed [153].

Regarding the quality of plant protein, it is known that all plant foods contain all twenty amino acids, including the nine essential amino acids; however, the amino acid distribution profile appears to be less optimal than that of animal foods [153]. In grains, the amino acid lysine is present in lower-than-optimal proportions for human needs and, likewise, the sulphur amino acids methionine and cysteine are proportionally lower in legumes than would be optimal. However, the terms “complete” and “incomplete” used to describe vegetable proteins are misleading because, especially in developed countries, plant proteins are mixed [153,154]. Theoretically, a totally plant-based diet oriented towards the consumption of cereals alone could lead to a lysine deficiency; however, in a plant-based diet, it is also possible to obtain a significant amount of total protein from a high intake of low-protein foods such as vegetables and fruits [155].

Evidence regarding a marked difference in plant protein digestibility in humans is very scarce. For example, the bioavailability of soy or pea protein isolates and wheat and lupine flour was quite similar to that of eggs (89–92% versus 91%) and meat (90–94%), and only slightly lower than that reported for milk (95%) [155]. It is also important to note that most of the plant proteins studied came from sources that are raw and unheated or minimally heated. These are the worst conditions for plant protein availability because of the presence of trypsin inhibitors and poor digestive enzyme accessibility [155].

Plant protein-rich foods such as traditional legumes, nuts, and seeds are sufficient to achieve full protein adequacy in adults that follow a vegetarian or vegan diet, while concerns about any amino acid deficiency have been substantially overstated. Intake of lysine might only be reduced in vegan individuals who have a low protein intake and who base their diet on a very limited range of foods, and where protein intake comes only from grains [156,157].

In nephropathy patients, two studies have demonstrated that a vegetarian LPD with a protein prescription of 0.70–0.75 g per kg of body weight could meet the reported requirements for all essential amino acids in the absence of a deterioration in nutritional status [89,98]. Previous studies have shown that when metabolic acidosis is corrected and energy intake is adequate, patients with advanced-stage CKD can fully adapt to low-protein regimens, and that patients can maintain a neutral or slightly positive N balance with a protein intake as low as 0.55–0.6 g/Kg [101]. Similarly, even the very-low-protein diet (0.3–0.4 g/kg/day, supplemented with keto-analogues to reach a protein intake of 0.55–0.6 g/kg of body weight/day) does not seem to be correlated with the onset of malnutrition, and does not lead to an increase in mortality in patients who subsequently start dialysis [158,159]. A more recent modelling study compared the supply of amino acids of a traditional LPD (comprising at least 50% animal protein) with the PLADO diet (only 30% animal protein) and a vegetarian and vegan LPD. At a protein prescription of 0.6 g/kg/day, only the conventional LPD was able to obtain the RDA (recommended daily allowance) for all essential amino acids. Instead, with a protein prescription ≥0.7 g/kg/day, all of the plant-based and vegetarian LPDs were able to obtain the RDA for all EAAs [122]. Regarding this study, other characteristics of vegetable-based diets (stronger in the vegan diet) were the higher content of vegetable fibre and the higher alkalizing power of the diet, which are both known to improve the nutritional status and protein metabolism of nephropathy patients through reducing intestinal-derived uremic toxins (in the case of fibre). This produces better adherence to diet and caloric intake as well asreduces the risk of metabolic acidosis, which has a deleterious effect on muscle metabolism [122,160].

Another study suggested that a higher fibre intake in nephropathy patients can reduce the levels of proinflammatory factors, indoxyl sulphate, and serum cholesterol, and is negatively associated with cardiovascular risk but does not disrupt the nutritional status of patients with CKD [161].

However, according to Garibotto and colleagues, plant proteins appear less anabolic than animal proteins in a low-protein diet, and plant proteins contain amino acids that in part may be oxidized. Thus, they may not be completely used for protein synthesis [162]. In particular, at lower protein intakes (10% of energy), animal proteins stimulate muscle protein synthesis more effectively than plant sources; on the contrary, at a higher protein intake, both animal and plant proteins cause similar anabolism, both in young and elderly patients [163].

The proposed strategies to improve the nutritional value of vegetable proteins were creating diets containing different plant protein sources to provide a high-quality AA profile, adding small quantities of animal protein to the diet, supplementing with essential amino acids or keto-analogues (KA), and consumption of greater amounts of plant protein [162]. Regarding KA supplementation in LPD, an RCT suggested a possible role not only in supporting nutrition status, but also in improving the correction of FGF-23 and Klotho abnormalities that may result in cardiovascular calcification [164]. Another possible strategy to improve the amino acidic and nutritional profiles of plant-based low-protein diets is to plan a diet based on a wide variety of plant foods, which should also include pseudocereals (good sources of AA, and particularly leucine, which appears to be critical for the stimulation of muscle protein synthesis), legumes (including soya, which presents a high nutritional profile), and nuts [165,166,167]. Regarding the intake of nuts in CKD patients, which is often prohibited for fear of an excessive intake of phosphorus and potassium, a recent randomized crossover trial showed that 30 g of walnuts per day in this population led to reduced blood pressure, LDL cholesterol, and albumin excretion, but had no effect on the physiological levels of phosphorous, potassium, PTH, and FGF23 [168]. In plant-based diets, oilseeds (especially walnuts and flaxseeds) are also an important plant source of omega-3 [31].

**Figure 2:**
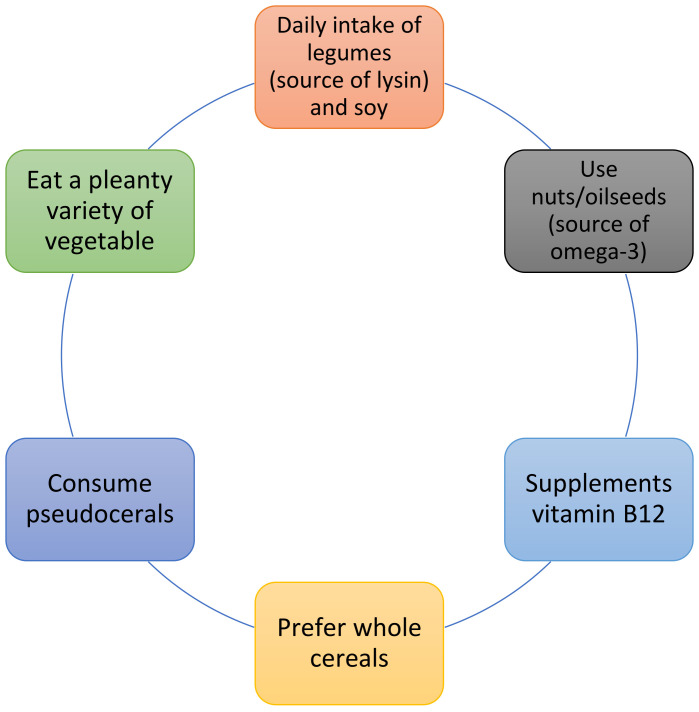
Strategies to improve the nutritional value of a plant-based diet.

To improve the nutritional value of the diet, whole grains should be consumed instead of refined grains. In fact, whole grains have great nutritional and bioactive properties because their fractions, bran and germ, contain unique health-promoting bioactive components and provide more B vitamins and fibre. Moreover, their phosphorus content is present as phytate, which is not digestible in the human gut, and so does not contribute to dietary phosphorus load [169,170] (Figure 2).

A recent umbrella review of whole grain consumption in the general population also found convincing evidence of an inverse association between whole grain consumption and the risk of type-2 diabetes, colorectal cancer, and cardiovascular mortality [171].

Unfortunately, the plant foods included in the diet of nephropathy patients are often monotonous because different limitations are imposed in relation to the risk of hyperkalaemia.

### 7.2. Hyperkalaemia

Hyperkalaemia is another challenge faced in prescribing a plant-based diet to CKD patients; currently, the approach to this problem is changing.

First of all, emerging data indicate that dietary potassium may be beneficial for patients with CKD. Epidemiological studies have shown that a higher urinary potassium excretion (which correlates with higher dietary potassium intake) is associated with lower blood pressure and lower cardiovascular risk in CKD patients, as well as better kidney outcomes; it seems that increasing dietary potassium may be equally as important as reducing sodium. In fact, recent studies have revealed that dietary potassium modulates the activity of the thiazide-sensitive sodium-chloride cotransporter in the distal convoluted tubule and promotes sodium excretion [172,173].

Moreover, there is limited and inconclusive evidence regarding the association between dietary potassium intake and serum potassium in patients with CKD; most of the observational studies that have tried to correlate serum potassium levels with estimated potassium intake based on a dietetic diary did not find any correlations [174]. Only one recent study has found a weak correlation between dietary potassium intake (estimated by multiple 24-h urine collections) and serum potassium levels [175].

Despite a lack of conclusive evidence, restricting dietary potassium has historically been recommended for CKD patients to prevent hyperkalaemia, which can cause severe arrhythmia, leading to death. However, restricting dietary potassium (which often means a reduction in healthy plant-based foods) can deprive individuals of the potential benefits of high dietary potassium intake for kidney function decline, end-stage kidney disease, and cardiovascular disease events [176].

Moreover, serum potassium levels and the prevalence of hyperkalaemia were not different between patients on an animal-based low-protein diet and a plant-based low-protein diet [177]. Similarly, Joshi and colleagues analysed prospective observational and experimental studies of nephropathy patients consuming varying proportions of plant-based diets and found only one patient with hyperkalaemia (due to a pre-existing type IV tubular renal acidosis and the consumption of raw edamame, which is one of the most potassium-rich foods) [178].

This evidence seems to suggest that dietary potassium intake may not be equivalent in terms of the effects of potassium among different foods. For example, the intake of potassium in foods of animal origin is associated with a simultaneous intake of acid load, which can predispose individuals to the development of metabolic acidosis and consequent hyperkalaemia. On the contrary, the intake of potassium from plant foods is associated with an alkalizing effect and with the intake of fibre (which reduces the risk of constipation) and carbohydrates (which stimulate the release of insulin and the consequent entry of potassium into the cells), all effects that reduce the risk of developing hyperkalaemia [170,179].

For this reason, currently, the indication to reduce the intake of potassium with diet is a strategy that is suggested only in the presence of documented hyperkalaemia and after excluding and correcting other possible causes (metabolic acidosis, uncontrolled diabetes, and constipation). Furthermore, the first foods to be restricted are those both rich in potassium and unhealthy (fruit juices, potato chips, chocolate, sauces, etc.) [178,180].

Finally, if hyperkalaemia develops, new potassium-binding medications (patiromer and sodium zirconium cyclosilicate) should be considered, rather than the healthy plant-based diet being discontinued [181].

### 7.3. Vitamin B12

Another possible concern regarding the use of plant-based diets in CKD patients is vitamin B12 (or cobalamin) deficiency. It is known that vitamin B12 deficiency is a growing problem, not only in plant-based diet consumers, but also in the general population, especially elderly people [182].

Supplementation with vitamin B12 in particular appears essential to plant-based diets, since this vitamin of bacterial synthesis is only contained in animal products.

However, all patients with CKD are at risk of deficiency of this vitamin due to reduced absorption (age reduces absorption capacity), due to the low intake of animal products in a low-protein diet, and due to the simultaneous intake of drugs, which can compromise the assimilation of vitamin B12 (proton pump inhibitors, metformin, etc). It must be taken into account that the ranges of this vitamin that are considered ‘normal’ have recently increased, so the optimal level should be greater than 488 pg/mL (however, often, laboratories indicate much lower normal ranges). Lower values correlate, in fact, with pathological levels of some indicators (methyilmalonic acid and holotranscobalamin), which suggest a metabolic deficit of B12 that can lead to vascular damage, neuropathy, and cognitive disorders [183]. According to Spence and colleagues, supplementation with cobalamin in CKD patients should be carried out using methylcobalamin or hydroxycobalamin instead of cyanocobalamin. In fact, cyanocobalamin can be toxic in kidney failure because patients with CKD have high plasma levels of tyocianate and cyanide, and additional cyanide from cyanocobalamin may accumulate with toxic effect [184].

### 7.4. Iron Deficiency

A systematic review found that iron intake in vegan diets is higher than in non-vegans, but this is not always reflected in ferritin levels due to low bioavailability [185]. In the general population, women who do not menstruate and men have the same prevalence of iron deficiency when following an omnivorous or vegetarian diet [186]. An observational study evaluated lacto-ovo-vegetarian and omnivorous patients with CKD stage 3–5 and found no difference in haemoglobin levels between the two groups [187]. Finally, an aforementioned modelling study that evaluates the nutritional intake of different low-protein diets also confirms that the plant-based low-protein options (vegan, ovo-vegetarian, and PLADO) meet the iron RDA for men and women [122]. Therefore, iron supplementation in patients with CKD should be individualized, regardless of the diet being followed.

## 8. Plant-Based Food Alternatives: Friend or Foe?

With the growing interest in plant-based diets, the market in recent years has manufactured a number of processed plant-based foods as an alternative to animal-based foods. Processed plant-based foods represent an interesting opportunity to simplify and vary the diet of CKD patients, especially within a plant-based low-protein diet. In general, plant-based products have a high content of fibres. Plant-based alternatives provide an average of 3.5–5 g of fibres per 100 g, which is absent in regular meat. However, some of these products, such as desserts, alternatives to cheese, ice cream, and ready meals do not seem to be healthy, both due to their high salt, sugar, and fat (mostly unsaturated) contents, and to their high content of preservatives [188]. Regarding these products, it is necessary to pay attention to their servings and frequency of consumption (as well as for the corresponding products based on animal foods) in order not to exceed recommended amounts, especially with regards to additives and salt intake.

On the contrary, plant-based meat alternatives (PBMAs), especially those based on pulses, may represent an important tool to increase the consumption and the palatability of legumes in the general population as well as in low-protein diets, and may offer similar nutritional benefits and reductions in chronic disease as whole legumes [189]. Another advantage of PBMAs is that certain components of legumes (e.g., oligosaccharides) that can cause flatulence are greatly reduced when the protein is extracted from the beans, thereby also improving digestibility [190].

A comparison between meat and PBMAs was made in the clinical trial Study With Appetizing Plant Food–Meat Eating Alternative Trial, wherein 36 participants consumed about 2.5 servings of PBMAs or analogous meat products daily, providing 25% of their total calories and 50% of their total protein, for 8 weeks. The results showed that consumption of PBMAs significantly decreased circulating levels of trimethylamine oxide (TMAO) and low-density lipoprotein cholesterol, as well as body weight, when compared with the meat products [191]. Moreover, PBMAs could serve as a vehicle for the fortification of nutrients that may be of concern when replacing animal protein with plant protein, such as iron, B12, and zinc [191]. Furthermore, research indicates that plant-based burgers have a low environmental footprint [192]. Regarding plant-based dairy substitutes, coconut milk may be the best dairy substitute for patients with CKD, based on low potassium, sodium, and oxalate [193].

## 9. Conclusions

As we have seen, the definition of “plant-based diets” encompasses different dietary patterns that share the trait of an increased intake of plant foods and the limitation or absence of animal foods. It would be useful to have a more precise definition of these diets, especially in terms of the amounts of animal products and ultra-processed foods that they allow. An ideal plant-based diet to prevent chronic kidney disease would probably contain minimal or no animal foods (to reduce acid load and phosphorus), and minimal or no processed unhealthy plant foods (to reduce sodium, phosphorus from preservatives, and empty calories). Most studies (including meta-analyses, umbrella reviews, and the position papers of scientific societies) have agreed that such diets are healthy, suitable for each phase of the life cycle, and able to prevent some pathologies such as hypertension, diabetes mellitus type II, and obesity. Since these pathologies currently represent the main cause of the onset of kidney damage, such diets seem useful in the primary prevention of CKD. Observational studies agree with the protective effect of such diets on kidney function. Furthermore, the features of plant-based diets (high fibre content, low acid load, poor bioavailability of phosphorus, low sodium content, better lipid profile) show the biological plausibility of their nephroprotective action. However, it would be useful to carry out randomized controlled trials in patients with early chronic kidney disease to evaluate the effective impact of a plant-based diet on kidney function and cardiovascular prevention. In patients with more advanced CKD (eGFR < 60 mL/min), it is possible to offer a low-protein plant-based diet (a vegan LPD supplemented or not supplemented, and a vegan VLPD supplemented). Evidence from RCTs supports the role of VLPDs in delaying dialysis, mainly through better control of uremic status (reduced levels of uremic toxins, improved mineral metabolism and acidosis), and in maintaining good nutritional status. Therapeutic adherence remains a fundamental requirement for the use and the effectiveness of the VLPDs; therefore, patients must be correctly selected. Unfortunately, most randomized controlled trials have compared the effects of traditional LPD with VLPDs; therefore, it is difficult to discriminate whether the benefit comes from the well-conducted strict protein restriction or from the vegetarian source of protein. Even though plant-based low-protein diets seem to be superior to mixed diets and could improve some metabolic disturbance, further studies are necessary to better investigate the possible metabolic and cardiovascular advantages of plant-based LPDs versus conventional LPDs. Regarding the possible concerns over the use of plant-based diets in nephropathy patients, it is important that these diets are planned with the help of a dietician to optimize the nutritional intake of plant foods and to minimize the risk of nutritional deficiencies and hyperkalaemia. We suggest, then, some strategies for implementing the nutritional value of plant-based low-protein diets: choose whole grains, use pseudocereals, encourage the intake of legumes, nuts, and soy, and ensure a vitamin B12 supplementation. However, clinical studies are necessary to verify the good tolerability and real impact of these strategies on nutritional parameters in nephropathy patients. The use of plant-based meat alternatives may also help to increase legume intake, as well as the palatability and nutritional value of plant-based diets for these patients. Specialist nutritional planning is certainly important in order to improve diet adherence and to optimize the foods eaten in terms of variety and nutritional quality. It would be also useful for dietitians to be trained on plant-based diets.

## Figures and Tables

**Table 1:** Observational studies analysing sources of protein, plant-based dietary patterns, and CKD incidence or prevalence.

Type of Study and Reference	Tool of Assessment	Time of Follow-Up	Population	Outcomes	Results/Bias
Observational prospective study, Kim H. et al., 2019 [29]	Food-frequency questionnaire	24 years	14.686 middle-aged adults	Incident CKD	Higher adherence to healthy plant-based diets and a vegetarian diet was associated with lower risk of CKD.
Observational cross-sectional study, Hao-Wen Liu et al., 2019 [41]	Food-frequency questionnaire	Cross-sectional	55.113 general population	Prevalent CKD (eGFR < 60 mL/min/1.73 m^2^ or proteinuria)	CKD was significantly less common in the vegan group compared with the omnivore group. Possible bias: selection bias, self-report, no portion size.
Observational retrospective study, Chia-Lin Wu 2023 [42]	Structured questionnaire of dietary habits (grouped into vegans, lacto-ovo-vegetarians, and omnivores)	11 years	3.618 patients with hyperuricemia	Incident CKD (proteinuria or eGFR < 60 mL/min/1.73 m^2^)	A vegan diet is associated with a 31% lower risk of CKD in patients with hyperuricemia.
Observational prospective cohort study, Asghari G. et al., 2017 [84]	Food-frequency questionnaire	6 years	1630 participants free of CKD	Incident CKD (eGRF < 60 mL/min/1.73 m^2^)	Adherence to the DASH-style diet was found to be inversely associated with incident CKD.
Observational prospective cohort study, Rebholz CM et al., 2016 [43]	Self-reported dietary intake different foods (evaluated with DASH diet score)	23 years	14.882 general population free of CKD	Incident CKD (eGRF < 60 mL/min/1.73 m^2^)	High red and processed meat intake was associated with risk of CKD; inversely, high intake of nuts, legumes, and low-fat dairy (high DASH diet score) was associated with reduced risk. Possible bias: self-reported dietary intake; lack of albuminuria.
Observational cohort study, Minesh Katri et al., 2014 [44]	Food-frequency questionnaire (Medi score)	15 years	900 general population	Incident CKD (eGRF < 60 mL/min/1.73 m^2^)	A Mediterranean diet was associated with a reduced incidence of CKD.
Observational retrospective study, Yi-Chou Hou et al., 2022 [85]	Structured questionnaire of dietary habits (grouped into vegans, lacto-ovo-vegetarians, and omnivores)	11 years	2.797 diabetic patients	Incident CKD (proteinuria or eGFR < 60 mL/min/1.73 m^2^)	Vegetarians and lacto-ovo-vegetarians had a lower incidence of CKD than the omnivores. Possible bias: self-report, diagnosis of diabetes mellitus based only on glycated haemoglobin.
Observational cross-sectional study, Vukovic Vladimir et al., 2023 [50]	Food-frequency questionnaire	Cross-sectional	5.889 free of hypertension, diabetes, or CKD	Serum creatinine (SCr) and eGFR	Total daily protein intake and daily protein intake from specific animal sources were positively associated with SCr and negatively associated with eGFR. Possible bias: related to muscle mass metabolism.
Observational prospective study, Quan-Lan et al., 2017 [56]	Food-frequency questionnaire	5 years	63.257 general population	End-stage renal disease	Red meat intake strongly associated with ESRD risk in a dose-dependent manner. Possible bias: self-report and one-time assessment of diet.
Observational prospective study, Parvin Mirmiran et al., 2020 [57]	Food-frequency questionnaire	6 years	4.881 general population free of CKD	Incident CKD	Higher consumption of total red meat and processed meat was associated with increased risk of incident CKD. Possible bias: self-report.
Observational prospective cohort study, Bernhard Haring et al., 2017 [59]	Food-frequency questionnaire	23 years	11,952 subjects free of diabetes and CVD, with eGFR> 60 mL/min/1.73 m^2^	Incident CKD	Red and processed meat consumption was associated with increased CKD risk; nuts, low-fat dairy, and legumes were found to be protective against the development of CKD. Possible bias: self-report.
Observational cross-sectional study, Oosterwijk Milou et al., 2019 [76]	Food-frequency questionnaire	Cross-sectional	420 diabetic type 2 patients	Prevalent CKD	Higher intake of vegetable protein was associated with a lower prevalence of CKD in DM type 2. Possible bias: protein intake was based on self-reported results.
Observational cross-sectional study, Lei Yin et al., 2023 [77]	Food-frequency questionnaire	Cross-sectional	20.733 rural adults	Prevalent CKD	Participants in the higher quartiles of bean intake had a lower prevalence of CKD. Possible bias: self-report.
Observational prospective study, Bernier-Jean Amelie et al., 2021 [83]	Food frequency questionnaire and eGFR at baseline, 5 and 10 years	10 years	1.374 Caucasian elderly women	eGFR decline	Higher intakes of plant-sourced protein were associated with slower declines in eGFR. Possible bias: self-report.

DASH: Dietary Approaches to Stop Hypertension. eGFR: estimated Glomerular Filtration Rate. CVD: Cardio Vascular Disease. DM: Diabetes Mellitus.

**Table 2:** RCTs on plant-based low-protein diets in non-dialysis-dependent chronic kidney disease (NDD-CKD).

Type of Study	Patients and Time	Comparing Diets	Results	Possible Bias
Prospective randomized controlled trial (Milovanova LY et al., 2023) [113]	85 CKD 3b-4 stages (12 months)	Soy LPD + KA versus traditional LPD + KA	Soy LPD + KA showed: - Slower decrease in eGFR; - Lower increase in left ventricle hypertrophy and better blood pressure control; - Better maintenance of lean body mass; - Improved urea and phosphorus and cholesterol levels.	
Prospective randomized controlled clinical study (Feiten SF et al., 2005) [114]	24 patients eGFR < 25 mL/min (4 months)	VLPDs versus conventional LPD	- Both maintain the nutritional status; - Only VLPDs showed improvement in calcium and phosphorus metabolism and a reduction in serum urea nitrogen.	
Prospective multicentre randomized controlled study (Brunori G. et al., 2007) [115]	56 uremic non-diabetic patients older than 70 years with eGFR of 5 to 7 mL/min (median follow-up was 26.5 months)	VLPDs versus dialysis	- VLPDs allowed for the postponement of dialysis for about 11 months without increasing mortality.	
Prospective randomized crossover-controlled trial (Di Iorio BR et al., 2018) [116]	60 patients on CKD stage 3b-4 (18 months)	Free diet (FD), Mediterranean diet (MD), and VLPDs	MD and VLPDs versus FD showed: - Lower diastolic blood pressure; - Lower urea, sodium, phosphorus and PTH; - Higher serum levels of bicarbonate and haemoglobin; - Lower lysine and homocitrulline/lysin ratio (markers of cyanate levels).	
Prospective randomized controlled trial (Garneata L et al., 2016) [117]	207 non-diabetic adults with stable eGFR < 30 mL/min (18 months)	VLPDs versus conventional LPD	Patients on a VLPDs showed: - Reduced risk of halving initial eGFR or dialysis initiation; - Lower serum urea and serum phosphorus; - Reduced need of calcium, bicarbonate, and vitamin D supplements.	
Prospective randomized controlled trial (Bellizzi V et al., 2022) [119]	233 patients (diabetics included) with CKD stage 4 or 5 (36 months)	VLPDs versus conventional LPD	VLPDs resulted in safe nutrition but without any benefit with regard to renal survival and metabolic parameters.	Low adherence to diet (only 3 patients followed the prescribed 0.3 g/kg/day of protein intake)

LPD + KA: Low Protein Diet plus Keto Analogues. eGFR: estimated Glomerular Filtration Rate. LPD: Low Protein Diet. VLPDs: Very Low Protein Diet supplemented.

**Table 3:** Different types of low-protein diet.

Diet	CKD Stage	Protein	Carbohydrates	Phosphorus
Conventional LPD	3–4	0.6 g/kg/day (>50% of animal origin)	Especially from low-protein substitutes	<700 mg/die
LPD Vegan	3–4	0.7 g/kg/day (100% from grain and legumes)	From cereals	
LPDS Vegan	3–4 Indicated in pregnant woman or in patients at high risk of malnutrition or in patients that do not tolerate legumes	0.6 g/kg/die (100% from cereals and legumes) + EAA/KA (1 tablet every 10 kg of body weight)	From cereals	
PLADO Diet	3–5	0.6 g/kg/day (50–75% from plant origin)	From whole cereals	
VLPDS	4–5	0.3–0.4 g/kg/die + EAA/AK (1 tablet every 5 kg of body weight)	Especially from low-protein substitutes	300–400 mg/die

LPD: low-protein diet, LPDS: low-protein diet supplemented, PLADO: plant-dominant low-protein diet, VLPDS: very low-protein diet supplemented. EAA/AK: essential amino acid/keto acid [101,110].
